# The thorium isomer ^229m^Th: review of status and perspectives after more than 50 years of research

**DOI:** 10.1140/epjs/s11734-024-01098-2

**Published:** 2024-01-31

**Authors:** Peter G. Thirolf, Sandro Kraemer, Daniel Moritz, Kevin Scharl

**Affiliations:** Faculty of Physics, Ludwig-Maximilians-Universität München, Am Coulombwall 1, Garching, 85748 Germany

## Abstract

Today’s most precise timekeeping is based on optical atomic clocks. However, those could potentially be outperformed by a nuclear clock, based on a nuclear transition instead of an atomic shell transition. Such a nuclear clock promises intriguing applications in applied as well as fundamental physics, ranging from geodesy and seismology to the investigation of possible time variations of fundamental constants and the search for dark matter. Only one nuclear state is known so far that could drive a nuclear clock: the “Thorium Isomer” ^229m^Th, i.e., the isomeric first excited state of ^229^Th, representing the lowest nuclear excitation so far reported in the landscape of nuclear isotopes. Indirectly conjectured to exist already in 1976, decades of experimental efforts were dedicated to unambiguously identify this elusive nuclear state and to characterize its properties. However, for 40 years, these efforts remained inconclusive. The turning point was marked by the first direct detection of ^229m^Th via its internal conversion decay branch in 2016. Since then, remarkable progress could be achieved in characterizing the properties and decay parameters. The half-life of the neutral isomer was determined, the hyperfine structure was measured via collinear laser spectroscopy, providing information on nuclear moments and the nuclear charge radius and also the excitation energy of the isomer could be directly determined with different techniques. In a recent experiment at CERN’s ISOLDE facility, the long-sought radiative decay of the Thorium isomer could be observed for the first time via implantation of ($\beta$-decaying) ^229^Ac into a vacuum-ultraviolet (VUV) transparent crystal and subsequent fluorescence detection in a VUV spectrometer. Thus, the excitation energy of ^229m^Th could be determined with unprecedented precision to 8.338(24) eV, corresponding to a wavelength of 148.71(42) nm. These achievements, together with ongoing laser developments for the required VUV wavelength, open the door toward a laser-driven control of the isomeric transition and thus to the development of an ultra-precise nuclear frequency standard.

## Introduction

Already back in 1976, Kroger and Reich conjectured the existence of an extremely low-lying first excited state in ^229^Th from their $\gamma$-spectroscopic measurements [1] targeting the nuclear structure of this light actinide isotope with a half-life of 7880 years. They could hardly anticipate the now almost 5 decades of worldwide efforts to be dedicated to this by far energetically lowest-lying nuclear excitation observed in the whole landscape of almost 3400 presently known isotopes with more than 186,000 excited states. Initially placed only coarsely within a range of 100 eV above the ground state and tentatively characterized as an isomeric M1 excitation from the $I^{\pi } = 5/2^+$ ground state to a $I^{\pi } = 3/2^+$ excited state, over the years, the excitation energy was refined first in 1990 from an estimate below 100 eV to $1 \pm 4$ eV and in 1994 to $3.5 \pm 1$ eV. A paradigm change was initiated in 2007, when improved detector technology led to a corrected energy value of 7.6(5) eV, now placing the thorium isomer in the spectral range of vacuum ultraviolet (VUV). Shortly after this value was revised to the for more than 10 years adopted value of 7.8(5) eV, corresponding to a VUV wavelength of $160 \pm 10$ nm [2–5].

**Fig. 1:**
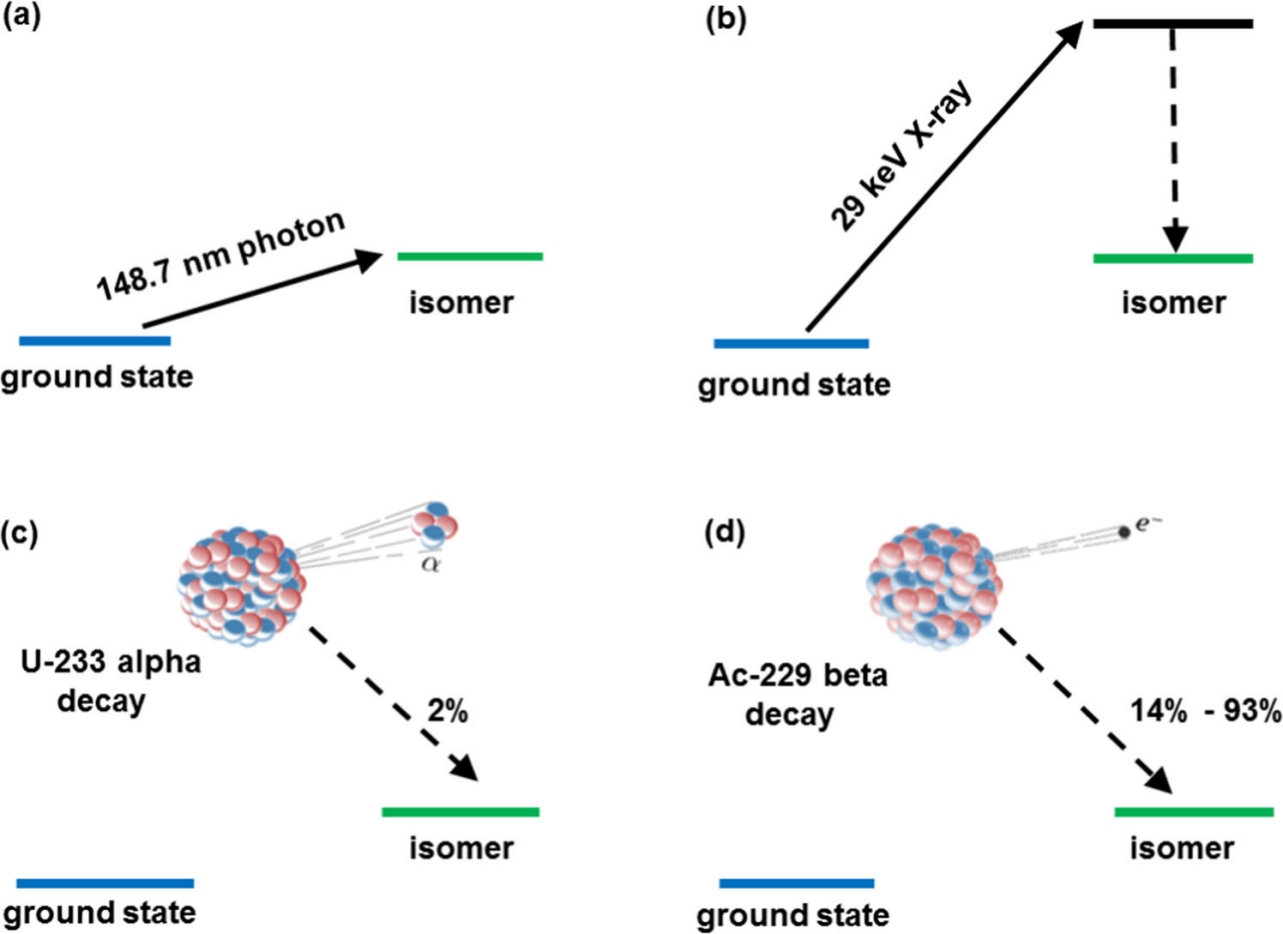
Different approaches for the population of ^229m^Th. **a** Direct resonant (laser-)optical excitation with (laser-)photons of suitable VUV wavelength (the value indicated in the panel will be motivated later on). **b** Indirect pumping of the isomer via the second excited state of ^229^Th located at ca. 29 keV via corresponding X-rays from a synchrotron facility. **c** $\alpha$ decay from ^233^U or **d** $\beta$ decay from ^229^Ac

While previous optical efforts to detect the isomer’s decay remained unsuccessful due to the wrong part of the spectrum being investigated with inappropriate instrumentation, the result by Beck et al. triggered various efforts toward a direct observation of the ^229m^Th ground-state decay, aiming at a precise determination of the excitation energy. Most commonly used methods were (VUV) fluorescence detection from ^229^Th-doped crystals and optical excitation in trapped ^229^Th ions [6–15]. These efforts were accompanied by proposals of various excitation and de-excitation schemes [16–19], preparations for laser-spectroscopic studies [20, 21], and quantitative assessments of the available parameter space for half-life and excitation energy studies [22]. Figure 1 sketches four approaches to populate the thorium isomer: (a) via direct resonant (laser-)optical excitation with a suitable laser wavelength (the value indicated in the panel will be motivated later on), (b) indirect pumping of the isomer via the second excited nuclear state of ^229^Th located at ca. 29 keV via corresponding X-rays from a synchrotron facility, (c) via $\alpha$ decay from ^233^U or (d) via $\beta$ decay from ^229^Ac.

**Fig. 2:**
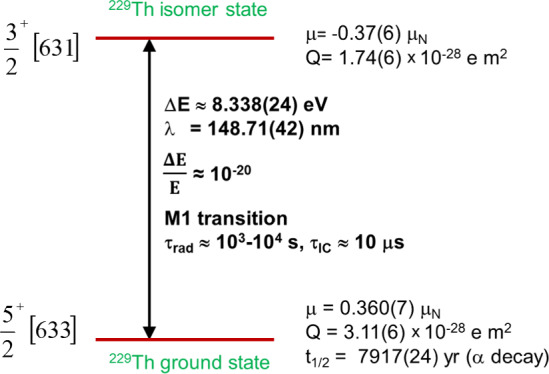
Illustration of our present knowledge of the nuclear level properties (excitation energy, lifetimes, relative linewidth, nuclear moments (magetic dipole, electric quadrupole) of the almost-degenerate ground-state doublet in ^229^Th (characterized by its spin-parity and Nilsson quantum number assignments). Updated from [23]

However, for 40 years, the elusive thorium isomer escaped direct identification via its ground-state decay and it was only due to several breakthroughs in recent years that we have now reached quite advanced insights, as will be detailed later in this article. Figure 2 illustrates our present knowledge of the thorium isomer’s basic properties, comprising its excitation energy and corresponding wavelength, radiative lifetime and nuclear moments.

A comprehensive historical literature overview from the first conjecture of ^229m^Th to its final direct detection in 2016 is given in the review article [24].

### ^229m^Th as novel nuclear frequency standard in a nuclear clock

It took about 15 years from its early conjecture to realize the potential of the thorium isomer for a variety of applications. Although Helmer and Reich never proposed any applications for ^229m^Th, in particular their publication from 1990 [2] sparked worldwide increasing interest, both theoretical and experimental. This led to the proposal of a broad scope of compelling applications in the subsequent years. Directly responding to [2], Strizhov and Tkalya in 1991 published a theoretical paper, where they discussed different decay channels of the thorium isomer [25]. Even more noteworthy, they already anticipated an increasing interest in the properties of ^229m^Th from various physics disciplines like “optics, solid-state physics, lasers, plasma, and others”.

Early discussions about the potential use of ^229m^Th for frequency metrology date back to 1996, when the “development of a high-stability nuclear source of light for metrology” was discussed by Tkalya et al. as an application for ^229m^Th [26]. The first detailed concept and analysis of a “nuclear clock” was worked out in 2003 by Peik and Tamm [27], addressing both an ion-based as well as a solid-state-based approach of its realization.

**Fig. 3:**
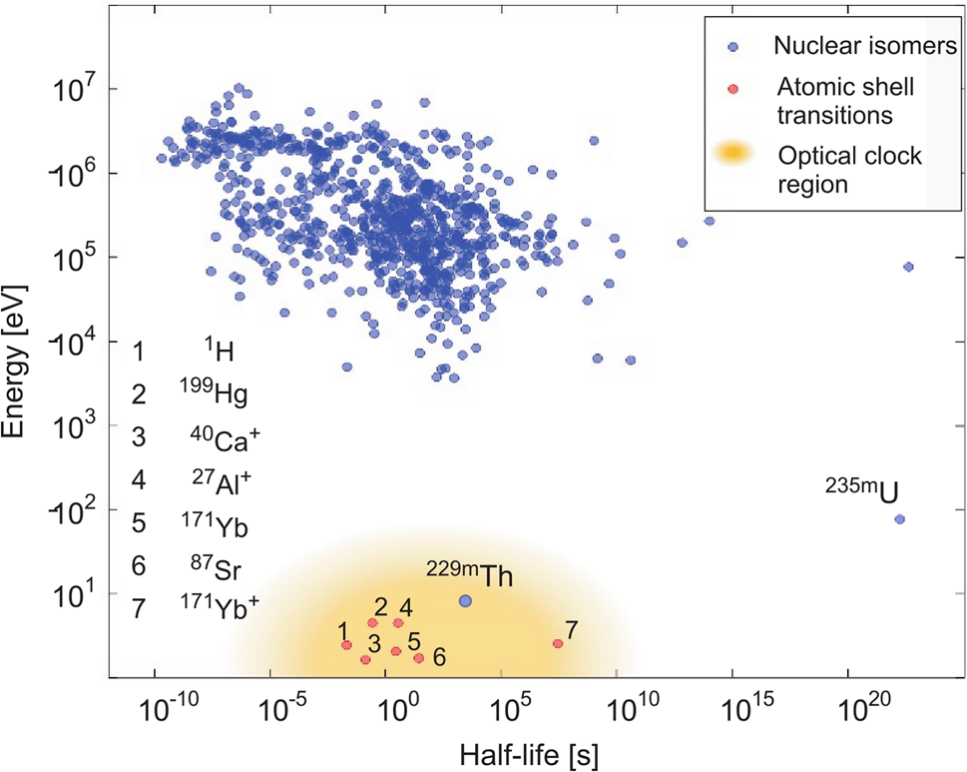
Distribution of excitation energy versus half-life of isomeric nuclear states. Nuclear excitations (blue circles) exhibit typical energies in the range of a few 10 keV up to several MeV. There are only two low-energy ($\le$1 keV) nuclear states known: ^229m^Th ($\sim$ 8.3 eV, expected energy range indicated by blue box) and ^235m^U (76.7 eV). Due to the very long radiative lifetime of ^235m^U, only ^229m^Th qualifies for a direct laser excitation, and hence for the development of a nuclear clock. In addition, selected clock transitions are included (red circles), which are already in use for optical atomic clocks. Updated and reprinted by kind permission from Springer: L. v.d. Wense et al., Nature 533, 47 (2016)

The uniqueness of ^229m^Th within the landscape of all presently known nuclear isomers as well as the proximity to established atomic clock transitions becomes evident in Fig. 3, where a 2D plot of excitation energy versus half-life for all presently known nuclear isomers is shown [28]. The thorium isomer is located far off all other excited isomeric nuclear states in the neighborhood of typical atomic transitions, which are applied in optical atomic clocks.

The advantages of applying a nuclear instead of an electronic shell transition are obvious: in conventional optical atomic clocks, the most significant perturbations from external-field induced clock frequency shifts arise due to differences in the clock state’s electronic wave functions. Due to the smallness of the nucleus as compared to the atomic electron cloud, leading to an excellent isolation of the nucleus from potentially perturbing external fields, the nuclear ground-state transition from ^229m^Th may be utilized in an optical clock of unprecedented accuracy and stability. Thus a nuclear clock can be expected to be largely resilient against external perturbations [27, 29–31]. Ultimately, a nuclear clock inaccuracy approaching the 10^-20^ scale (in [29] a total clock uncertainty budget of 1.5$\cdot$10^-19^ is estimated for a ^229m^Th based nuclear clock) appears viable with current ion clock technologies applied to a ^229^Th^3+^ nuclear clock system [29]. This has to be seen in the context of the rapid parallel development of atomic clocks, where the presently best optical atomic clocks exhibit total systematic frequency uncertainties as low as 9.5$\cdot$10^-19^ [32–35].

Two main approaches have been proposed for the realization of a nuclear clock based on ^229^Th: one based on trapped ions and another one using doped solid-state crystals. The first approach starts from individually trapped Th ions, e.g., in a (cryogenic) Paul trap, comparable to trap-based optical atomic clocks. This approach promises an unprecedented suppression of systematic shifts of the clock frequency and leads to the above-mentioned expected nuclear clock uncertainty of about 10^-19^ [29]. According to [29], the remaining systematic error budget for a ^229^Th^3+^ nuclear clock using single-ion-clock technologies will be dominated by excess micromotion in the trap and gravitational shifts, while, depending on the achievable power provided by the VUV clock laser, power broadening and phase noise will add to the intrinsic linewidth. The other approach relies on embedding ^229^Th in vacuum-ultraviolet (VUV) transparent crystals (e.g., CaF_2_, MgF_2_, LiSrAlF_6_ or LiCaAlF_6_) [27, 31, 36, 37]. This bears the advantage of the large number ($\ge 10^{18}$ cm^-3^ has already been reached) of Th nuclei included in the crystal, leading to a considerably higher signal-to-noise ratio and a higher stability of the nuclear clock [38]. The achievable uncertainty will be limited in this case by systematic crystal effects. In [38], a detailed study of the interaction between the thorium nuclear levels and the crystal-lattice environment is presented, which can cause inhomogeneous effects that will affect the performance of a solid-state nuclear clock. The magnetic dipole interaction and the second-order Doppler effect are identified as the main sources of inhomogeneous line broadening, while static global line shifts are expected to be dominated by the temperature-dependent electric monopole and electric quadrupole interactions and by second-order Doppler shifts, necessitating active temperature stabilization of the sample.

In all scenarios, a suitable, narrow-band VUV spectroscopy laser is required for the excitation of the thorium isomer from the ground state of ^229^Th. Therefore, a precise characterization of the thorium isomer’s properties (especially the nuclear ^229m^Th transition) with laser-spectroscopic precision remains a mandatory prerequisite for any kind of nuclear clock.

**Fig. 4:**
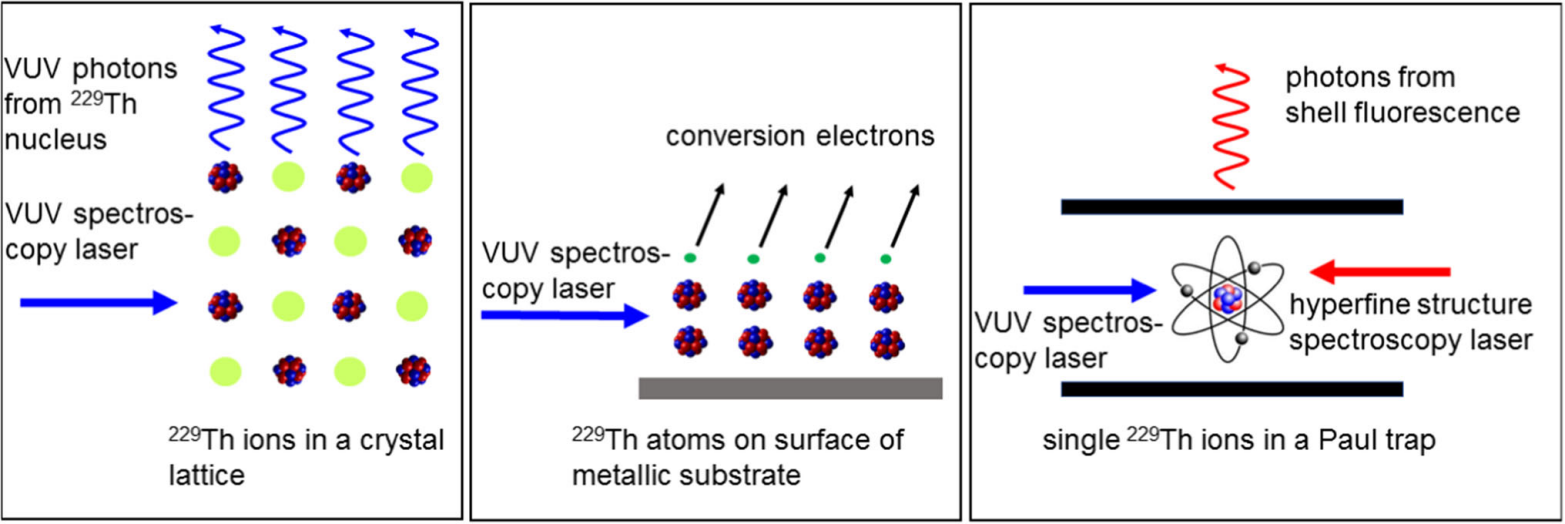
Different approaches to realize a nuclear optical clock: left: crystal-lattice nuclear clock, middle: internal-conversion nuclear clock, right: single-ion nuclear clock. Adapted from [24]

Figure 4 illustrates the two above-mentioned approaches (left panel: solid-state approach, right panel: ion trap approach), together with an alternative scenario, where neutral ^229^Th atoms are deposited on the surface of a metallic substrate, thus enabling internal conversion decay (middle panel).

A review of the ideas and concepts for a frequency standard that is based on a radiative transition in an atomic nucleus rather than in the atomic electron shell can be found in [24, 35, 39].

### Fundamental and practical applications of a nuclear clock

Similar to other ultra-precise optical clocks, a nuclear clock could become a tool to address a variety of practical as well as fundamental physical applications. Moreover, due to its different operational principle, being sensitive not only to the electromagnetic and weak force, but also to the strong interaction, a nuclear clock can be regarded a novel type of quantum sensor beyond its timekeeping properties.

Precision clocks play a decisive role in developing increasingly accurate satellite-based navigational systems like the GPS system [40] or similar existing or upcoming systems worldwide. Present systems are limited by position uncertainties in the meter range. Realizing an envisaged future accuracy in the (sub-)cm range by use of ultra-precise clocks (like state-of-the-art atomic lattice clocks or a future nuclear clock) would open up new horizons for a variety of applications like autonomous driving, freight or component tracking and many more (see articles compiled in [41]).

In the framework of general relativity, geometry and gravity cannot be treated independently and relativistic effects gain more and more relevance in modern geodesy (“relativistic geodesy”) [42, 43]. High-precision clocks are suitable tools for relating the gravitational potential to an atomic (or future nuclear) frequency reference [44]. The relative relativistic “gravitational red-shift” of the clock frequency $\Delta f/f$ is related to a variation $\Delta U$ of the gravitational potential via $\Delta f/f = \Delta U/c^2$ with *c* as the speed of light. A relative frequency shift of $10^{-18}$ (representing the accuracy of the presently best atomic optical clocks) corresponds to a height difference of 1 cm or a sensitivity to geopotential differences of $\Delta U = 0.1$ m^2^ s^-2^. An expected improvement in the future by about an order of magnitude would, e.g., provide millimetric sensitivity to a network of synchronized ultra-precise (atomic or nuclear) clocks for local modifications of the gravitational potential. Thus, monitoring of the filling of volcanic magma chambers or early-detected plate tectonic movements may come into reach as practical applications for a future nuclear clock based on the thorium isomer [45, 46]. The gravitational potential on Earth oscillates with a period of 1 year due to the ellipticity of the Earth’s orbit. Therefore, searching for a variation of clock frequency ratios during the year also serves as a test for the validity of Einstein’s equivalence principle (see Sect. II.I of [47]).

As outlined in [23], with different sensitivities to specific effects of new physics beyond the Standard Model, meaningful comparisons can be made between a clock system of high sensitivity and a stable reference of low sensitivity. The ^229^Th nuclear clock would be an attractive addition to the already presently used ensemble of atomic clocks used in fundamental physics tests, because it will provide a strongly enhanced sensitivity for variations of fundamental constants. The possibility of temporal and spatial variations of fundamental constants like the fine-structure constant $\alpha$ and the scale parameter of the strong interaction m_q_/$\Lambda _{\textrm{QCD}}$ is suggested in several theories that unify gravity with other interactions [47, 48]. Following an idea introduced by Peik and Tamm in 2003 [27], Flambaum was the first in 2006 to quantitatively assess the potential of the ^229m^Th ground-state transition to enhance the sensitivity of measuring the relative effects of potential temporal variations of $\alpha$ and $m_{q}$/$\Lambda _{\textrm{QCD}}$ [49] by several orders of magnitude. This sensitivity enhancement factor is given as the ratio $K= {{\Delta ~V_{\textrm{C}}} \over {\omega }}$ between the Coulomb energy difference of ground state and isomeric first excited state in ^229^Th and the excitation energy $\omega$ of the thorium isomer. The factor *K* can be determined from measurements of the mean-square charge radii and electric quadrupole moments of the ground and first excited states of ^229^Th [50]. However, current measurements of the electric quadrupole moment [51] are yet to achieve an accuracy high enough to resolve nonzero sensitivity. In a recent study, the enhancement factor was estimated as $K = - \left( 0.82 \pm 0.25\right) \cdot 10^4$ [52]). If experimentally confirmed, it would pave the way for a determination of time-dependent variations of fundamental constants and, consequently, for all related searches for new physics, such as searches for dark matter (DM).

Nuclear clocks also could become instrumental in the search for ultralight dark matter [39]. Despite a variety of observational evidence for the existence of non-luminous dark matter, i.e., a massive component of the universe that does not absorb, reflect, or emit any type of electromagnetic radiation, no clear experimental signature identifying or constraining its nature could be achieved so far [53–59]. Various theories suggest dark matter candidates in a mass range spanning more than 78 orders of magnitude, ranging from ultralight scalar fields (10^-21^ eV to about 1 eV) up to 30 solar masses (10^58^ eV) from black hole observations. In the regime of massive dark matter, decades of search for “weakly interacting massive particles” (WIMPS) in the mass range of MeV to TeV so far only provided exclusion boundaries. An alternative to heavy-particle dark matter is so-called ultralight dark matter (ULDM), i.e., a class of dark matter models (DM) where DM is composed by bosons (scalar fields) with masses ranging from 10^-21^ to $\le$ 1 eV. Axions (hypothetical elementary particles with electric charge 0 and spin 0) and topological defects (monopoles, 1D strings, 2D domain walls) are potential candidates for ultralight DM [60, 61].

In our Galaxy, ultralight scalar dark matter fields exhibit coherence behaving like a wave with an amplitude, where $\rho _{\textrm{DM}} =$ 0.3 GeV/cm^3^ is the local DM density [62]. The coupling of such DM to the Standard Model leads to oscillations of fundamental constants and, therefore, clock transition frequencies. Such an oscillation signal would be detectable with atomic and nuclear clocks for a large range of DM masses and interaction strengths [63–65]. Ultralight topological defect fields may cause variations in the fundamental constants of nature (in particular the fine structure constant $\alpha$), which may in turn lead to shifts of atomic energy levels. Such shifts could be monitored via high-precision atomic or nuclear clocks. Therefore, it has been proposed to use synchronized networks of high-precision clocks as dark matter sensors for transient signals of topological dark matter. Even more stringent limits on topological DM properties can be derived from non-transient variations of fundamental constants via the back action of ambient matter. Such back action produces an environmental dependence of the fundamental constants of nature as well as spatial variations of the fundamental constants in the vicinity of dense bodies such as Earth due to the formation of a ‘bubblelike’ defect structure surrounding the dense body [66].

There also exist proposals toward a nuclear $\gamma$-ray laser [67] (detailing the initial mentioning of this idea in [26, 68]) based on the ^229m^Th ground-state transition and a nuclear qubit for quantum computing [69].

Moreover, as already proposed for optical atomic lattice clocks, an ultra-precise nuclear clock based on the thorium isomer could serve as gravitational wave detector [70].

## Experimental identification and characterization of ^229m^Th

After its conjecture from $\gamma$ spectroscopic data in 1976 [1], it took 40 years until a first direct identification of the thorium isomer ^229m^Th could be achieved [71], allowing for subsequent further characterization of its basic properties and thus opening the door toward its future application as driver for a nuclear clock.

### Direct identification of the thorium isomer

**Fig. 5:**
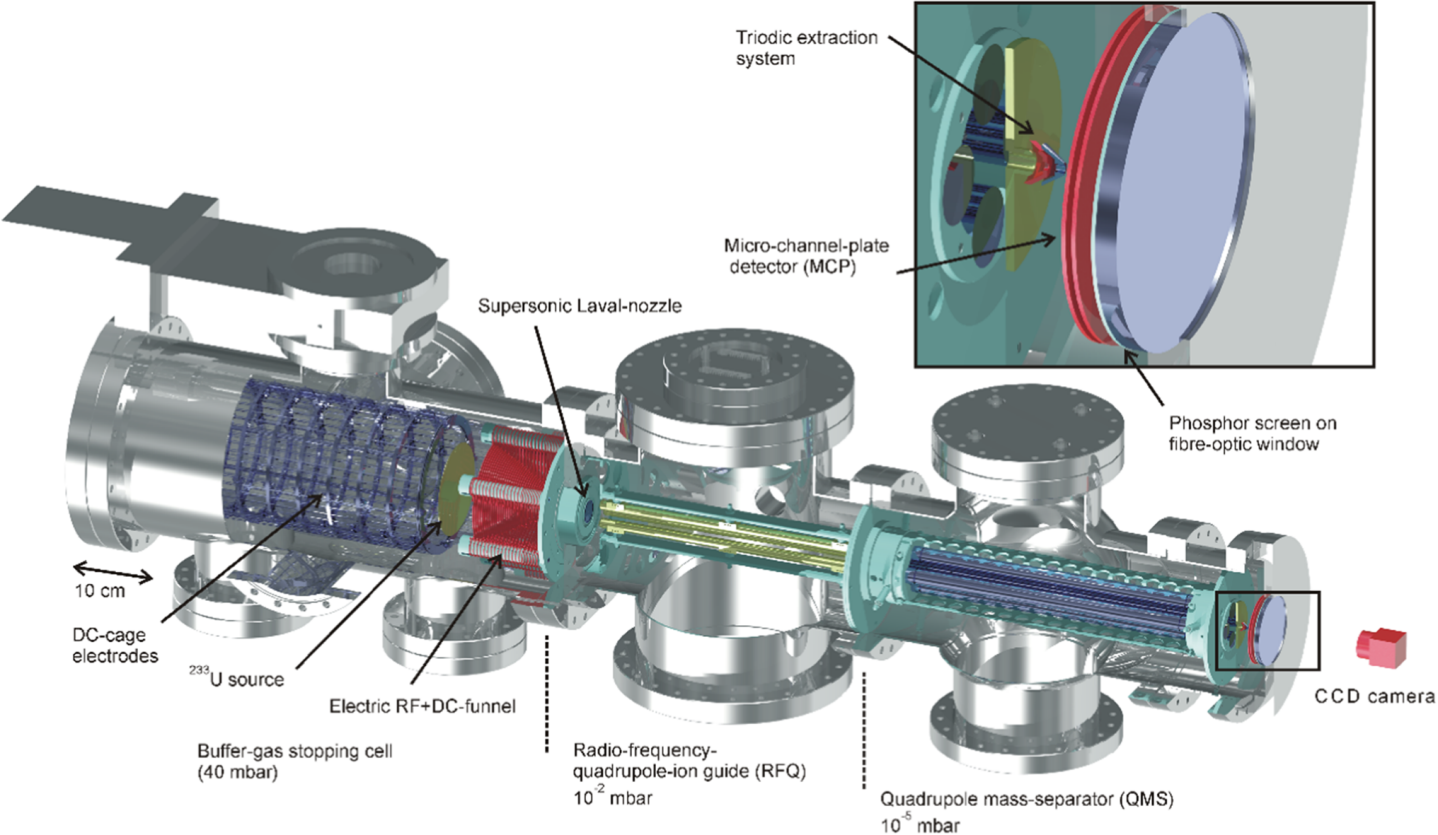
3D technical drawing of the experimental setup employed for the generation of an isotopic clean ^229(m)^Th ion beam. It is composed (from left to right) of a buffer-gas stopping cell that houses a ^233^U $\alpha$ recoil source, a radiofrequency quadrupole (RFQ) as ion guide and phase space cooler, a quadrupole mass separator (QMS) and (behind a triodic electrode system) a multichannel-plate detector followed by a phosphor screen and a CCD camera. For more details, see the main text [46]. Reprinted by kind permission from Springer: L. v.d. Wense et al., Nature 533, 47 (2016)

In these experiments, the “standard” method to populate the thorium isomer was chosen: $\alpha$ decay from ^233^U with its 2$\%$ decay branch into ^229m^Th (see Fig. 1c). To discriminate against prompt background during $\alpha$ decay, population and decay detection of ^229m^Th were spatially separated using a buffer-gas stopping cell setup [72] as shown in Fig. 5.

The $\alpha$-recoil ions, which possess a kinetic energy of up to 84.3 keV for ^229^Th, are stopped within 1–2 cm in 32 mbar of ultra-pure helium. A conical RF$+$DC funnel system, consisting of 50 ring electrodes, is employed to guide the ions through the buffer-gas toward the gas-cell exit, which is formed by a supersonic nozzle. In the region of the nozzle (0.6 mm diameter), a supersonic gas jet is formed that rips the ions off the electrical field lines and drags them with the gas jet into the subsequent vacuum chamber that houses a 12-fold segmented, radiofrequency quadrupole (RFQ) ion guide. In this extraction chamber, the He carrier gas is pumped away, leaving a selectable ambient pressure of 10^-2^–10^-3^ mbar for phase space cooling. The segmentation of the extraction RFQ can be used to operate it as a linear Paul trap, allowing for ion bunch formation. Subsequently the ions are transferred into a quadrupole mass separator (QMS), serving to filter out the ^229^Th ions of interest from their accompanying ^233^U decay chain daughter products. Finally, behind the QMS, the extracted ions are guided and focused by a triodic electrode structure, consisting of three ring electrodes in a nozzle-like shape. The total extraction time for ions from the gas cell amounts to a few ms and singly to triply charged thorium ions can be extracted. After failing to observe radiative VUV fluorescence de-excitation from extracted ions collected on a metallic catcher surface, the decay signal could for the first time be unambiguously identified in the internal conversion decay channel, which is energetically allowed as soon as charged ^229^Th ions are neutralized on a metallic surface (as then the isomer’s excitation energy is larger than the first ionization potential of thorium (ca. 6.3 eV). A clear signal of conversion electrons was observed, emitted during the decay of the thorium isomer on the surface of a multichannel-plate detector (MCP) and imaged by via a phosphor screen on a CCD camera. Confirmation tests included the use of a ^234^U source, whose $\alpha$ decay to ^230^Th did not result in any conversion electron signal, thus ruling out the atomic shell as origin of the observed signal [71].

More details of the methodology to generate the ^229m^Th isomer beam are described in [46], the corresponding visualization is contained in [73].

### Lifetime determination of neutral ^229m^Th

Following the direct identification of the thorium isomer’s ground-state decay, the natural next experimental steps focused on a characterization of its properties, e.g., its half-life. Here the segmentation of the extraction-RFQ was exploited to operate it as a linear Paul trap, allowing to create short ion bunches exhibiting a time-of-flight (TOF) width of about 10 $\mu$s and containing about 600–800 ^229(m)^Th ions in the 2^+^ or 3^+^ charge state, respectively. Due to the limited storage time in the Paul trap of about 1 min, a measurement of the ionic half-life, expected to amount to 10^3^–10^4^ s, was out of reach in the experimental setup. Instead, the half-life of the neutral isomer could be addressed using the same detection technique as before based on conversion electrons registered after neutralization of the ions in an MCP detector.

**Fig. 6:**
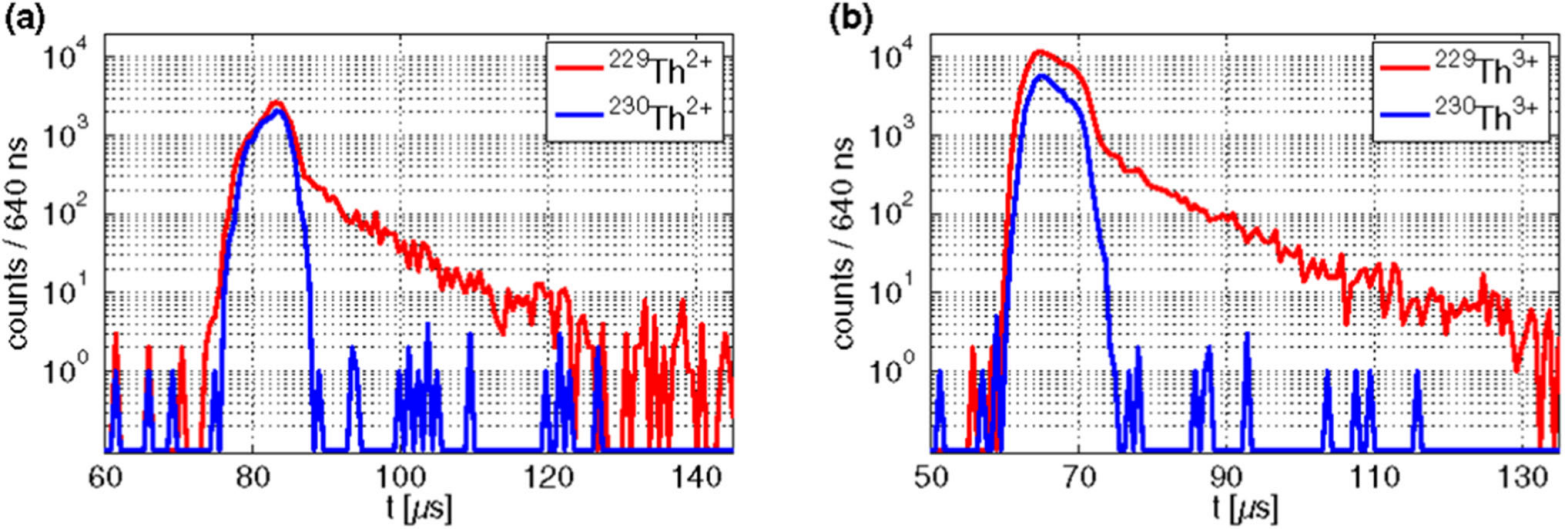
Measurement of the isomeric decay with a bunched **a** ^229(m^Th^2+^ and **b** ^229(m)^Th^3+^ ion beam (red curves). Corresponding comparative measurements performed with ^230^Th^2+^ and ^230^Th^3+^ are also shown as blue lines. Reprinted with permission from B. Seiferle et al., Phys. Rev. Lett. 118 042501 (2017). Copyright 2019 by the American Physical Society

Figure 6 displays comparative measurements with bunched (a) ^229(m)^Th^2+^ and (b) ^229(m)^Th^3+^ ion beams (red curves), together with data from ^230^Th^2+,3+^ ion bunches, extracted from a ^234^U $\alpha$-recoil source (blue curves). The absence of an exponential decay tail in ^230^Th ions confirms the origin of the exponential tail as resulting from the decay of the thorium isomer ^229m^Th [46, 74].

Half-lives of $t_{1/2} = 6.9 \pm 1.0\,\mu$s and $t_{1/2} = 7.0 \pm 1.0\, \mu$s were obtained for measurements performed with ^229(m)^Th^2+^ ions and ^229(m)^Th^3+^ ions, respectively. This half-life determined for the neutral thorium isomer agrees with the theoretically expected lifetime reduction in case of IC decay by about nine orders of magnitude [22, 75] based on an internal conversion coefficient $\alpha _{\textrm{IC}} =10^9$.

### Characterization of the hyperfine splitting of the thorium isomer

As with our increased knowledge on the properties of the thorium isomer, the realization of a nuclear clock built on ^229m^Th comes closer within reach, the question gains importance on how to identify a nuclear excitation from the ground to the isomer state provided a suitable laser would become available. A solution for this questions already dates back 20 years to a proposal of Peik and Tamm [27]. Laser excitation of the ^229^Th nucleus into the isomeric first excited state can be detected via a double-resonance method, analog to Dehmelt’s ‘electron shelving’ scheme for the detection of the excitation of metastable states in the atomic shell of individually trapped ions. The method builds on probing the hyperfine structure of a transition in the electron shell as described in [27] and [46]: a first laser (frequency $\omega _1$) is tuned on a closed two-level electric-dipole transition in the electronic shell, resulting in the emission of resonantly scattered fluorescence photons. In case of a successful excitation of the thorium isomer via a second laser with frequency $\omega _2$, the nuclear moments, the nuclear spin and hence the hyperfine splittings and total angular momenta of the electronic levels will change. This will in turn cause $\omega _1$ to fall out of resonance. The resulting drop of resonance scattering fluorescence intensity then serves as indicator of the nuclear excitation.

**Fig. 7:**
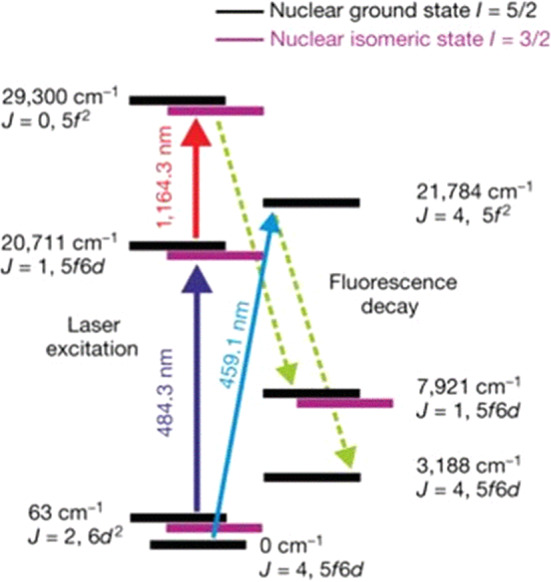
Transitions and electronic configurations of Th^2+^ levels relevant for the two-step collinear laser spectroscopy experiment performed to resolve the hyperfine splitting components of the thorium isomer. Reprinted by kind permission from Springer: J. Thielking et al., Nature 556, 321–325 (2018)

Exploit this technique requires knowledge of the hyperfine structure (HFS) of the thorium isomer. To determine the HFS of ^229m^Th, the method described in Sect. 2 to extract ^229(m)^Th ions from a ^233^U source was combined with a collinear, Doppler-free two-step laser spectroscopy excitation scheme $(J = 2 \rightarrow 1 \rightarrow 0)$ as indicated in Fig. 7 [51].

A first blue laser at 484 nm was applied to excite ions from a selected velocity class of the broad thermal line profile of the initial state ($J=2$, 63 cm^-1^) to an intermediate state ($J=1,$ 20,711 cm^-1^). This laser was detuned in steps of 120 MHz width across the thermal profile, while the intermediate state was probed by a second tunable laser at a wavelength around 1164 nm. Resonant excitations to the final state ($J=0$, 29,300 cm^-1^) were induced and for each of the 35 steps of the first laser, a frequency scan over 4 GHz was performed with the second laser to register the hyperfine structure resonances. Based on the nuclear ground-state spin $I=5/2$ of ^229^Th and $I=3/2$ for the thorium isomer, 9 (for the ground state) and 8 (for the first excited isomeric state) hyperfine splitting components were observed [51]. This allowed to extract the hyperfine constants A (representing the magnetic dipole interaction) and B (strength of the electric quadrupole interaction) from the equation

1 $$\begin{aligned}{} & {} E_{\textrm{HFS}}\left( JIF\right) = \frac{1}{2} AK + B \frac{(3/4)K(K+1)-I(I+1)J(J+1)}{2I(2I-1)J(2J-1)} \end{aligned}$$

with *I* as the nuclear spin, *J* as the total angular momentum, $F= I + J$ and $K = F(F+1) - J(J+1) - I(I+1)$ [76, 77]. From A and B, the nuclear moments can be derived: from the measured ratio $A^m/A =$ $-$1.73(25), the magnetic dipole moment $\mu ^m$ of the isomer was derived as $\mu ^m = \mu A^m/A I^m (AI)$, where $\mu$ indicates the magnetic moment of the ground state, while *I* and $I^m$ represent the nuclear spins of ground and isomeric states, respectively. The nuclear magnetic dipole moment $\mu$ of the ground state is known as $\mu =0.360(7)\mu _N$ with $\mu _N$ denoting the nuclear magneton [20, 78]. Hence, the magnetic dipole moment of the thorium isomer follows as $\mu ^m=$0.37(6)$\mu _N$. The spectroscopic electric quadrupole moment of the isomeric state $Q_s^m$ was determined from $Q_s^m= Q_s B^m/B$, where Q_s_ is the spectroscopic quadrupole moment of the ground state [20, 78, 79]. Thus, $Q_s^m$ follows as $Q_s^m =$ 1.74(6) eb [51]. From the spectroscopic quadrupole moment, the intrinsic quadrupole moment $Q_0$ can be derived as $Q_0^m=8.7(3)$ eb for the isomeric state and $Q_0= 8.8(1)$ eb for the ground state, respectively [51].

In addition, the difference of the mean-square nuclear charge radii of the isomeric first excited and nuclear ground state in ^229^Th could be determined in [51] as $\delta \langle r^2 \rangle = \langle r_{229\,m}^2 \rangle - \langle r_{229}^2 \rangle = 0.012(2)~fm^2$. Soon after, this value could be refined by new data on the ground state charge radius to $\delta \langle r^2 \rangle = 0.0105(13)~fm^2$ [80].

As indicated in Sect. 1.2, the sensitivity enhancement factor $K= {\Delta V_C \over \omega }$ for variations of the fine-structure constant $\alpha$ can be quantified based on the mean-square charge radii and electric quadrupole moments of ground and excited state in ^229^Th [50] according to

2 $$\Delta V_C = -485\,\text {MeV} \left[ {\langle r_{229m}^2 \rangle \over \langle r_{229}^2 \rangle } -1 \right] + 11.6\,\text {MeV} \left[ {Q_0^m \over Q_0^g} - 1 \right]$$

Despite having achieved a relative uncertainty of 4$\%$ for the electric quadrupole moment, the aforementioned experiment resulted in a value for the difference of the Coulomb energy of ground state and isomer in ^229^Th of $\Delta V_{\textrm{C}} =$ $-$0.29(43) MeV [51], as such still lacking sufficient precision to estimate *K* for the thorium isomer. Further laser experiments on laser-cooled ion samples will allow to improve the precision on $Q_0$, enabling a conclusive result.

### Determination of the excitation energy

The ‘holy grail’ on the road toward an optical control of the thorium isomer as prerequisite of realizing a nuclear clock is precise knowledge of the isomer’s excitation energy. In the last 4 years, significant progress on this key property was achieved with different techniques.

**Fig. 8:**
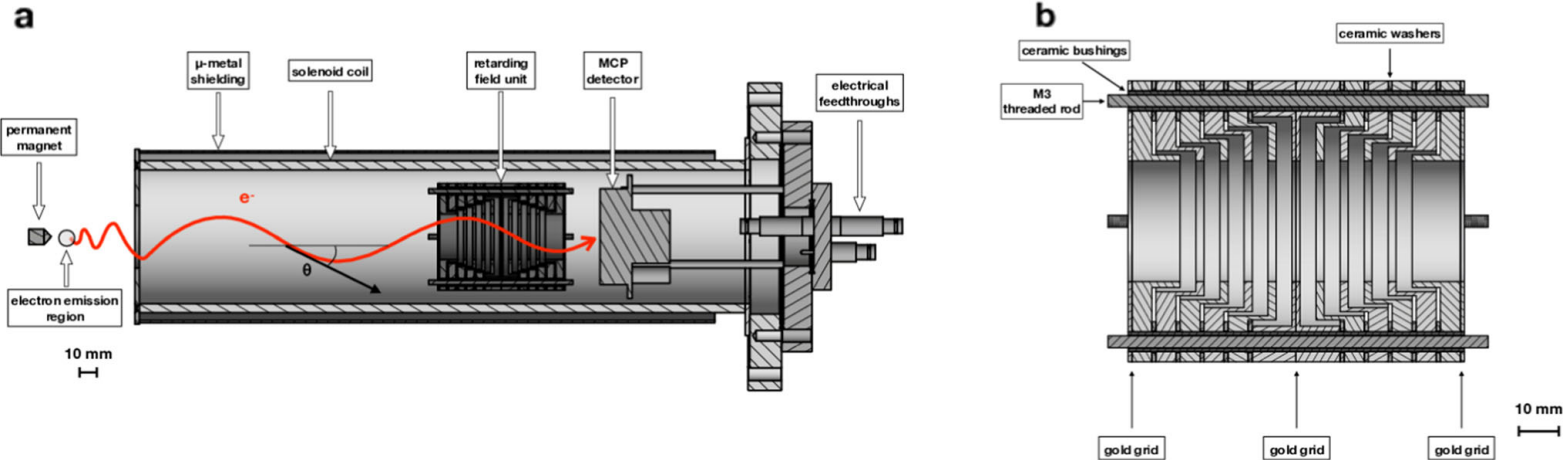
Sectional view of the magnetic-bottle type retardation electron spectrometer. The left panel (**a**) shows an overview of the spectrometer, with the permanent magnet, the solenoid coil, the retarding field unit, and the MCP detector. The region of electron emission as well as a helical electron trajectory with its pitch angle $\theta$ is indicated. The right panel (**b**) shows a detailed view of the retarding field unit. The positions of the gold grids are indicated by arrows. Reprinted from B. Seiferle et al., Nucl. Instr. Meth. A, 10.1016/j.nimb.2019.03.043, Copyright (2019), with permission from Elsevier

In a natural continuation of the IC-based ^229m^Th studies, the experimental setup described in Sect. 2.1 was complemented by a customized magnetic-bottle electron spectrometer. This enabled a determination of the kinetic energy of conversion electrons after neutralization of ^229m^Th ions in flight while traversing a graphene foil. The conversion electrons were collected in an inhomogeneous magnetic field region created by a strong permanent magnet and were subsequently focused into the solenoidal field of the electron spectrometer, which is shown in a sectional view in Fig. 8.

Electrical retarding fields are generated by a retarding field unit that consists of 12 concentric ring electrodes. Gold grids are sandwiched between the two central electrodes and are placed at the entrance and exit of the unit. The retarding voltage, ranging between 0 and $-10$ V, is applied to the central electrodes, while the entrance and exit electrodes are grounded. Only conversion electrons with kinetic energies larger than the retarding potential finally reach an MCP detector. Analyzing the resulting experimental data contains the challenge that resonant neutralization of ^229m^Th^q+^ ends in excited atomic states and that the subsequent IC decay leads to excited electronic final states. Therefore, theoretical guidance on the ensemble of relevant electronic levels was imperative in the extraction of the excitation energy of the thorium isomer $E^*(\text {iso})$ from the observed IC electron kinetic energies $E_{\textrm{kin}}(e)$ according to

3 $$\begin{aligned} E_{\textrm{kin}}(e) = E^*{(\mathrm iso)} - \text {IP} - E_{\textrm{ion,final}} + E_{\textrm{atom,initial}} \end{aligned}$$

with IP as the well-known first ionization potential of thorium (IP(Th) = 6.30670(25) eV, [81]). Finally, this analysis resulted in the first direct determination of the thorium isomer’s excitation energy, providing a value of $E^*(\text {iso}) = 8.28 \pm 0.17$ eV, equivalent to a VUV-optical wavelength of $\lambda = 149.7 \pm 3.1$ nm [82]. This value consolidated the localization of the nuclear resonance in an spectral region where no cw lasers are available at present. Therefore, this measurement also served as indicator for which laser technology to be selected when aiming at developing a driver laser for a future nuclear clock.

Soon after this study, groups from Heidelberg and Vienna published a complementary result from measurements of the low-energy (0–60 keV) $\gamma$-ray spectrum produced in the $\alpha$ decay of ^233^U using a dedicated cryogenic magnetic microcalorimeter (MMC) [83]. The MMC’s energy resolution of $\sim$10 eV allowed for determining the energy of the low-lying thorium isomer using four complementary evaluation schemes. The most precise of them determined the ^229^Th isomer energy to be 8.10(17) eV, corresponding to $\lambda =$ 153.1(32) nm, superseding in precision previous values based on $\gamma$ spectroscopy, and agreeing with the above described direct IC electron measurement.

A different experimental concept was applied at the Japanese SPring8 synchrotron facility. Optical pumping into ^229m^Th could be demonstrated, achieved using narrow-band 29-keV synchrotron radiation to resonantly excite the second excited state of ^229^Th. This will decay predominantly into the first excited state, the thorium isomer. The 29 keV nuclear resonance energy was determined with an accuracy of 0.07 electronvolts, a half-life of 82.2 ps could be measured together with an excitation linewidth of 1.70 neV. Also the branching ratio from the second excited state into the ground and isomeric state could be determined. These measurements allowed to constrain the ^229m^Th isomer energy (however with rather large uncertainties) by combining them with $\gamma$-spectroscopy data collected over the previous 40 years [84].

#### First identification of the radiative decay branch

While the direct ground-state decay of the thorium isomer via the IC decay branch could be observed and exploited, the radiative VUV de-excitation escaped experimental observation until recently. The situation changed with an experiment that did not use any more the $\alpha$ decay of ^233^U ($t_{1/2}= 160,000$ years) to populate the isomer, but instead the $\beta$ decay from ^229^Ac, a short-lived radio-isotope with $t_{1/2}= 62.7$ min. Thus, it needs to be produced in an online facility, as in this case the ISOLDE facility at CERN [85], where a high-energy proton beam impinges on a uranium target, causing spallation reactions. The resulting nuclides diffuse out of the target, get ionized, mass separated and acclerated to 30 keV. In a series of experiments, short-lived $A=229$ nuclides (Fr, Ra) were implanted into VUV transparent (large bandgap) CaF_2_ and MgF_2_ crystals, where the $\beta$-decay chain Fr $\rightarrow$ Ra $\rightarrow$Ac ultimately populated ^229^Ac [86]. Electron channeling was applied to prove that the implantation indeed largely ended in lattice positions, substituting host matrix atoms [87]. This represents a mandatory prerequisite for the occurrence of radiative decay of the thorium isomer, as an implantation into interstitial positions in the host crystal lattice would create electronic states in the band gap, in turn opening the internal conversion decay branch, and thus inhibit radiative decay.

**Fig. 9:**
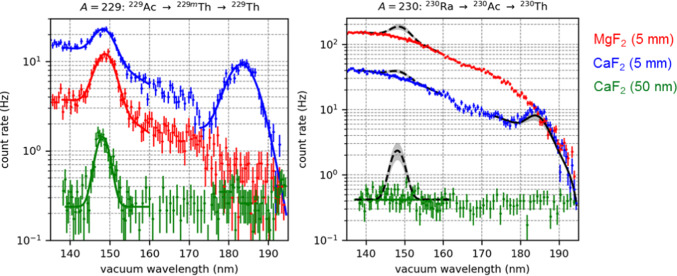
VUV photon emission spectra of crystals with implanted ^229m^Th ions, exhibiting a resonance at around 148.7 nm from the radiative decay of the thorium isomer. This resonance does not appear after implantation of ^230^Th. Reprinted by kind permission from Springer: S. Kraemer et al., Nature 617, 706–710 (2023)

Despite the effort to be invested in generating ^229^Ac, the advantages compared to the established method of using the $\alpha$ decay of ^233^U are obvious: the $\alpha$-decay recoil energy of ^229^Th from ^233^U amounts to about 84 keV. This rather violent decay process would largely affect the structure of ^233^U doped crystals, largely driving the dopants off their lattice positions, thus distorting the electron shell and potentially opening up the non-radiative IC decay channel. In contrast, the smooth $\beta$ decay of ^229^Ac with a recoil energy of less than 6 eV leaves the implanted crystal structure unaffected, thus enabling the radiative decay of the isomer. Moreover, the nuclear level scheme of ^229^Ac provides an about 7–47-fold higher branching ratio into the ^229m^Th isomer compared to ^233^U [86]. At ISOLDE, the implantated crystals were rotated from their irradiation position into an adjacent position in front of the entrance slit of a high-resolution, large aperture VUV grating monochromator, monitored by a photomultiplier tube. Repeatedly scanning the spectrometer’s diffraction grating provided the emission spectra shown for three different crystals in Fig. 9.

In all crystals, a clear signal at around 148.7 nm could be observed for the implantation of $A=229$ nuclei (left panel), while no such signal appeared when instead the same beam elements with $A=230$ were implanted (right panel). This unambigously proved for the first time the radiative decay of the thorium isomer. The measurements provided a sevenfold improved precision of the excitation energy, now determined as $E^*\left( ^{229m}\text {Th} \right) = 8.338(24)$ eV, equivalent to $\lambda = 148.71(42)$ nm [86]. From the decay of ^229m^Th embedded in a MgF_2_ crystal, also a decay curve and a related half-life could be derived, resulting in a value of $t_{1/2}=670(102)$ s. Taking into account the $n^3$ scaling (assumed here to be valid based on the electron channeling findings of dominant implantation to lattice sites in the bulk despite the shallow implantation depth) with the refractive index of MgF_2_ at 148 nm results in an estimate for the ionic half-life of the thorium isomer of about 2500 s, well within the range expected by theory. However, as already pointed out in [86], the validity of the $n^3$ dependence needs further investigation obtaining more precise half-life values and performing implantations under different conditions and in different crystals.

The breakthrough observation of the radiative isomer de-excitation demonstrates a proof-of-principle for the feasibility of a solid-state based nuclear clock.

## Perspectives toward the nuclear clock

**Fig. 10:**
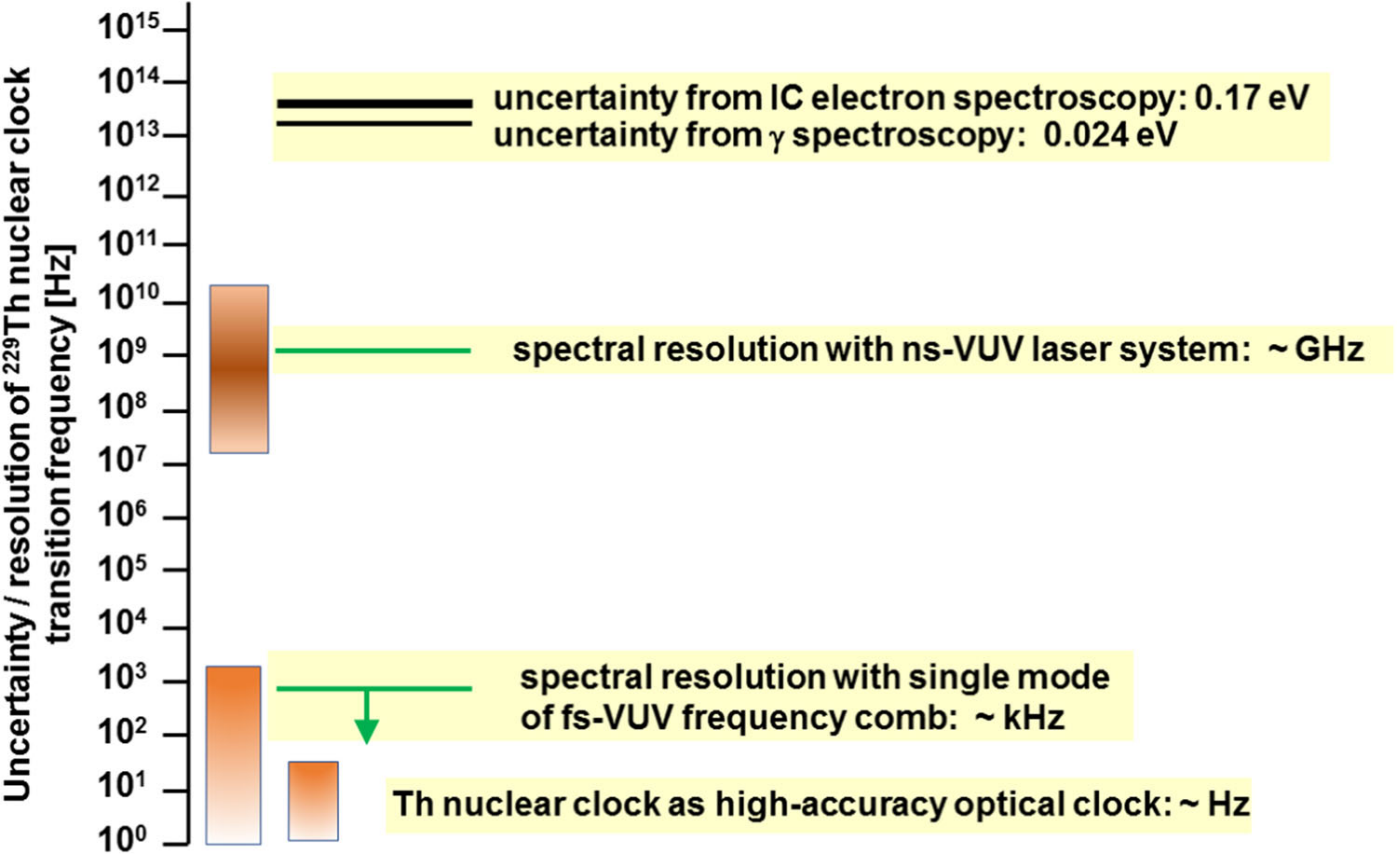
Overview of presently achieved (via nuclear spectroscopic methods) and ultimately (for precision clock operation) required frequency precision of the nuclear resonance in ^229m^Th. Between the present and targeted knowledge still about 12 orders of magnitude need to be bridged. This is envisaged in two steps, starting with broadband frequency scans using existing VUV laser system with few GHz bandwidth. In a second step, VUV frequency comb lasers will be used for narrow-band precision scans with $\le$ kHz bandwidth

With the level of quantitative insight into the properties of the unique thorium isomer ^229m^Th reached in the framework of intense efforts over the last few years, the now almost 50-year-old “thorium-229 story” has reached a pivotal point: from the nuclear physics-driven “search and characterization phase” to a laser-driven “consolidation and realization phase”. While on a nuclear physics scale, the precision of the excitation energy of the thorium isomer has reached already superb quality via conversion electron and $\gamma$-spectroscopy techniques, on a level relevant for laser spectroscopy and ultimately for metrological nuclear clock operation, we are still lacking many orders of magnitude and we have to make the transition from the energy scale in eV to a frequency scale in (k)Hz. This is illustrated in Fig. 10.

The presently most precise isomeric excitation energy reached via decay spectroscopy as discussed in Sect. 2.4.1 amounts to 2016 THz, with an uncertainty of about 6 THz. This is still about 10–12 orders of magnitude away from the metrologically relevant region of Hz–kHz as the resolution of the ^229^Th nuclear clock transition frequency, corresponding to fractional uncertainties to be improved from presently $10^{-3}$ down to a ‘start version’ of a nuclear clock with about $10^{-15}$ fractional uncertainty or ultimately $10^{-19}$ as envisaged for an ultra-precise nuclear clock.

**Fig. 11:**
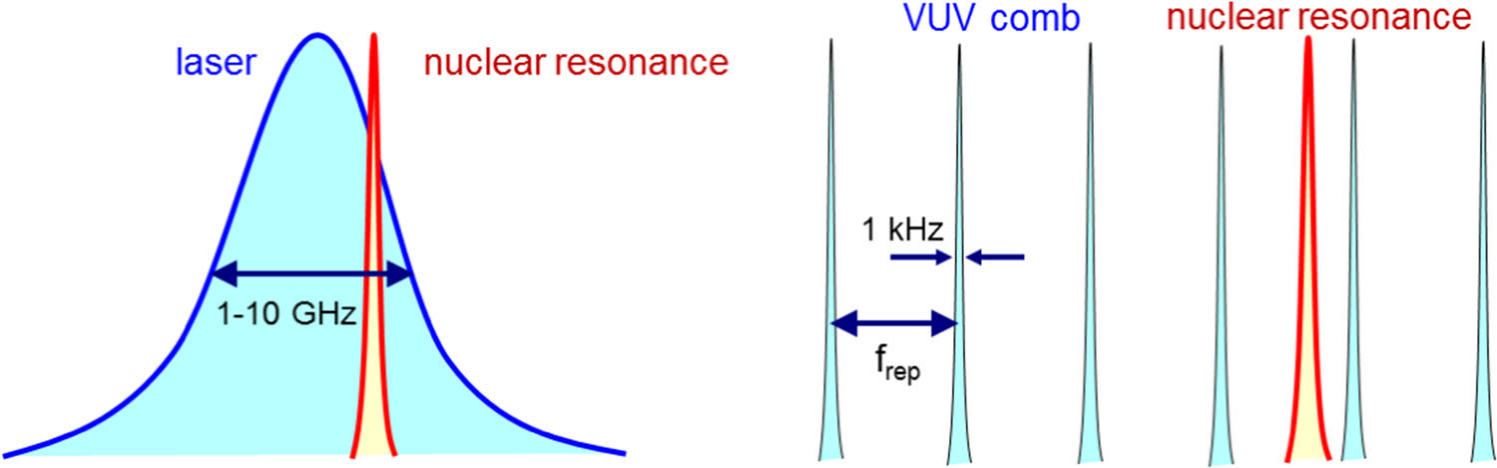
Complementary search strategies to excite the nuclear resonance of the thorium isomer via direct VUV laser spectroscopy. Left: scanning a broadband laser with few GHz bandwidth across the search region or (right panel) using a narrow-band VUV frequency comb where scanning the free spectral range inbetween two comb modes (e.g. 40–80 MHz) will result in one of the (typically few 10^5^) modes coming into resonance with the ^229m^Th nuclear level

So the next breakthrough has to come from an optical excitation of the ^229m^Th nuclear resonance with laser-spectroscopic precision. Already existing broadband VUV laser systems with bandwidths of a few GHz could achieve in a first step, the first optical excitation of the nuclear resonance, allowing to constrain the search range for subsequent scans with narrow-band lasers (about kHz bandwidth), like the VUV frequency comb described in Sect. 3.2. Figure 11 illustrates the two conceptually different search strategies for laser-spectroscopic identification of the nuclear ^229m^Th resonance. The before mentioned broadband search with already existing laser systems with GHz bandwidth is shown in the left panel. This procedure is adequate for an initial search as it allows to shorten the scan times while compromising on the frequency resolution. In contrast, narrow-band search with high frequency precision, yet in longer scan times (potentially already exploiting the constraint of the scan range by successful broadband excitation), can be achieved using a frequency comb [88] (right panel of Fig. 11). Here the scan over the full frequency range of the comb can be performed by only scanning the free spectral range inbetween two comb modes, as all other equally spaced modes will take part in the search process. Finally, strategies exist on how to identify the mode number responsible for the nuclear excitation once the search was successful.

### Toward the determination of the ionic lifetime of ^229m^Th

**Fig. 12:**
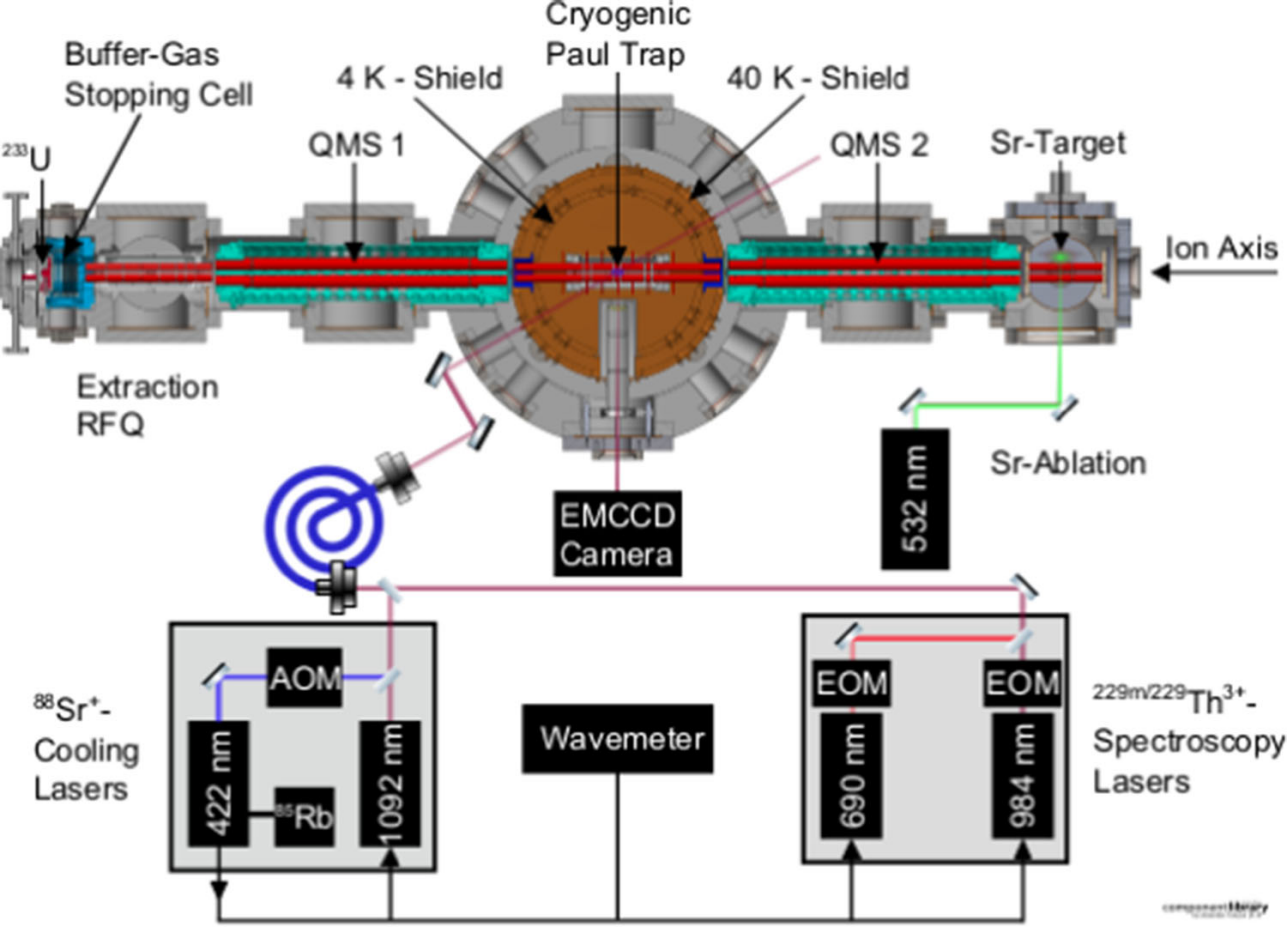
Schematic overview of the cryogenic Paul trap setup dedicated to ^229m^Th^3+^ radiative lifetime measurement. Visible is a horizontal cross-sectional cut of the trap vacuum chamber along the ion axis with a not-to-scale but logical arrangement of the different laser setup sections. From [89]

^229(m)^Th ions will be delivered to the central cryogenic Paul trap from by a compact buffer-gas stopping cell (filled with 32 mbar catalytically purified He gas), housing a 10 kBq ^233^U source together with guiding RF$+$DC funnel-shaped ring electrodes for the extraction of thermalized recoil ions through a de-Laval nozzle into a segmented radiofrequency quadrupole ion guide. Subsequently a purified ^229(m)^Th ion beam is formed in a quadrupole mass separator. This section is equivalent to the one introduced in Sect. 2.1. The opposite side of the cryotrap features another quadrupole mass separator, used to select the ^88^Sr^+^ ions sent from a laser ablation target towards the cryotrap.

To determine the last yet unknown basic property of the thorium isomer and to further specify the linewidth of its ground-state transition, a measurement of the ionic lifetime of the isomer is in preparation at LMU Munich. Theory and experimental investigations predict the lifetime to be 10^3^–10^4^ s, which is supported by the in-medium lifetime findings presented in Sect. 2.4.1. To precisely target this property using hyperfine structure spectroscopy, an experimental setup is currently being commissioned. It is based on a cryogenic Paul trap providing long-enough storage times for ^229m^Th ions that will be sympathetically cooled with ^88^Sr^+^. A sketch of the complete setup is shown in Fig. 12.

As detailed in [89] and shown in Fig. 12, the laser-optical setup comprises four continuous-wave diode laser systems and one pulsed Nd:YAG ablation laser. The latter serves to generate ^88^Sr^+^ ions which will be co-trapped with (Doppler-cooled at 422 nm) thorium ions in the linear Paul trap. Photons at 1092 nm wavelength will ensure efficient repumping from the metastable 4d ^2^D_3/2_ state. Quantitative details of the experimental setup are reported in [89].

The radiative lifetime of the isomeric state will be measured using hyperfine structure spectroscopy (HFS) at 690.32 and 984.19 nm to address the ^2^F$_{5/2} \rightarrow$ 6p ^2^D_5/2_ and ^2^F$_{7/2} \rightarrow$ 6p ^2^D_5/2_ transitions in ^229,229m^Th^3+^ [90] with natural linewidths of FWHM = 28 kHz and FWHM = 210 kHz, respectively [91].

The envisaged measurement scheme to determine the radiative lifetime of ^229m^Th^3+^ builds on using the HFS-related spectral differences between the nuclear ground state and the isomeric state as detailed in [90]. The cw lasers at 690 and 984 nm overlap with the trapped thorium ions and are either configured to address the electronic HFS of thorium ions in the ground state or to drive the HFS transitions of the isomeric state. By switching back and forth between the different settings, the detected fluorescence light either stems from ions in the nuclear ground-state or in the isomeric excited state [89, 90].

In a first step of the periodic lifetime measurement cycle, Sr ions will be loaded into the cryogenic Paul trap and laser-cooled until forming a Coulomb crystal. Subseqently, thorium ions are loaded from the opposite side and sympathetically laser cooled. In a second step, the fluorescence intensity emitted by the thorium ion(s) in the isomeric state will be registered with the 690 and 984 nm spectroscopy lasers in the respective wavelength configuration. At that moment, all thorium ions in the nuclear ground state will be ‘dark’. Directly after a recorded disappearance of the isomer (either by decay or loss), the spectroscopy lasers will be switched to only address the hyperfine structure transitions of the ground-state ions. This will allow to distinguish between nuclear decay or ion loss processes in the trap. Once identified as a valid decay event, the measurement cycle will be repeated to acquire sufficient statistics, finally allowing to derive the ionic lifetime as the last missing basic nuclear property of the thorium isomer.

### Toward an optical control of the ^229m^Th nuclear clock transition

**Fig. 13:**
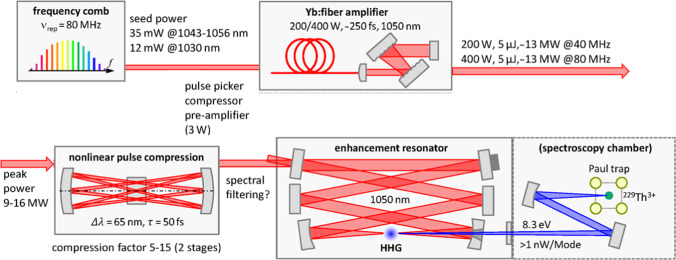
Layout of the components forming the VUV frequency comb presently under development as driver for exciting the nuclear clock transition in ^229m^Th. For details see the text

Constraining the excitation energy of the thorium isomer as described in Sect. 2.4 marked a major achievement also from a practical perspective in view of the ultimate need of an optical (i.e., laser-driven) control of the thorium isomer’s excitation for operation of a nuclear clock. It is now evident that presently no narrow-band and intense cw laser exists in the relevant wavelength regime around 148 nm. Instead the development of a ^229m^Th nuclear clock laser has to proceed based on different laser concepts. Such a laser system is presently under development at the Fraunhofer ILT (Institute for Laser Technology) in Aachen in the framework of the ThoriumNuclearClock ERC Synergy project [92]. Instead of a cw laser, here a VUV frequency comb is targeted, i.e., a pulsed laser system with a spectrum formed by a multitude of equally spaced individual lines. This technique provides the advantage that all comb modes can simultaneously contribute to the precision search for the wavelength of the nuclear ^229m^Th transition. Moreover, such pulses can provide a high pulse intensity, which is favorable when exploiting non-linear effects. Narrow-band lasers are only available in the infrared (IR) or visible spectral range. However, they can be converted to shorter wavelengths. In principle, this also works for continuous-wave (cw) lasers when using suitable non-linear crystals. However, for wavelengths below 205 nm, such crystals are not available. Therefore, the development of a ^229m^Th nuclear clock laser builds on a non-linear conversion process in a gas, called ’high harmonic generation’ (HHG). This process requires to start with a high intensity as only available from pulsed lasers. The required VUV wavelength of about 150 nm (with ca. 2 nm bandwidth) will be reached as the seventh harmonic of an infrared frequency comb at 1050 nm (operated at 40 MHz repetition rate). To reach sufficient power in the VUV regime despite the small conversion efficiency, the IR frequency comb will first be amplified to high power (200 W). Then the spectral range will be broadened, i.e., the pulse length will be compressed in a non-linear multi-pass pulse compression unit to about 50 fs before being sent into a resonator. The shorter pulses allow for a higher conversion efficiency, while the broadened spectrum is better suited for spanning the search range for the nuclear ^229m^Th resonance. In the resonator, the pulses will be enhanced to a circulating power of about 10 kW, driving the HHG generation in a xenon gas jet. Besides the power enhancement, the resonator also assists to preserve the narrow linewidths in the laser spectrum. A sketch of the building blocks of the laser system is shown in Fig. 13.

An average VUV comb power of $\ge$ 0.35 mW is expected (derived from 10 mW average IR comb power around 1050 nm), which is sufficient to excite the nuclear resonance if tightly focused onto the thorium ions trapped in the linear Paul trap. This laser system, envisaged to become operational in spring 2024, together with the previously introduced cryogenic Paul trap will finally form the platform to realize a first prototype of a nuclear clock based on the thorium isomer.

### Alternative excitation schemes

As seen before, direct VUV laser excitation of the ^229m^Th isomeric state requires a large effort of developing new laser concepts due to the lack of appropriate cw lasers in the relevant VUV wavelength regime around 150 nm. Alternatively, using relativistic ^229^Th ions in (high-energy) storage rings, high-power lasers with wavelengths in the visible range or longer can be used to achieve high excitation rates of ^229^Th isomers. The various options based on this concept have recently been reported in a review article [93]. High excitation rates can be realized through direct resonant excitation or excitation via an intermediate nuclear or electronic state, facilitated by the tunability of both the laser-beam and ion-bunch parameters. Unique opportunities are also offered by highly charged ^229^Th ions due to the nuclear-state mixing. The subsequently significantly reduced isomeric-state lifetime corresponds to a much higher excitation rate for direct resonant excitation. In [93], it is also for the first time proposed to use electric-dipole transitions changing both the electronic and nuclear states that are opened by the nuclear hyperfine mixing. It is suggested to using them for efficient isomer excitation in Li-like ^229^Th ions, via stimulated Raman adiabatic passage (STIRAP) or single-laser excitation. Ref. [93] also proposes schemes for probing the isomers, utilizing nuclear radiative decay or laser spectroscopy on electronic transitions, through which the isomeric-state energy can be determined with an orders-of-magnitude higher precision than the current value.

## Conclusion

Since its indirect conjecture in 1976, the elusive low-lying thorium isomer ^229m^Th has puzzled generations of nuclear physicists. In turn, its unique properties, foremost the lowest excitation energy so far observed in any nuclear isotope, have triggered numerous proposals for applications of ^229m^Th, both in the realm of fundamental physics as well as in practical areas. Most notably, the thorium isomer and its ground-state transition can be applied as the driving two-level system for a highly-precise and stable nuclear frequency standard, a ‘nuclear clock’. Being less vulnerable to external perturbing fields due to the smallness of the nuclear dipole and quadrupole moments, such a nuclear clock could potentially even outperform optical atomic clocks as the presently best timekeeping devices. Moreover, a nuclear clock represents a novel quantum sensor with high sensitivity for fundamental physics beyond the Standard Model of particle physics. In contrast to atomic clocks, also the strong interaction contributes to the frequency of a ^229m^Th-based nuclear clock, rendering sensitivity in the hadronic sector inaccessible with to atomic clocks. Hence, searches for ultralight scalar dark matter candidates can be conducted with a highly sensitive nuclear clock (in synchronized comparison with atomic clocks), as well as for signatures of variations of fundamental constants like the fine-structure constant $\alpha$ or the dimensionless strong interaction parameter $m_q \over \Lambda _{\textrm{QCD}}$ (with $m_q$ being the quark mass and $\Lambda _{\textrm{QCD}}$ the quantum chromodynamics (QCD) scale).

Despite these intriguing perspectives, it took 40 years until the first direct identification of the thorium isomer in the internal conversion ground-state decay branch. This breakthrough in 2016 sparked intense efforts of further characterization of the isomer’s properties, leading to determinations of the half-life of neutral ^229m^Th, the unveiling of the hyperfine structure of the thorium isomer and finally also precision measurements of the isomeric excitation energy with different techniques. Among them, the most recent breakthrough, where the so far still missing radiative decay branch could be identified via VUV fluorescence spectroscopy following implantation of *beta*-decaying ^229^Ac nuclides into VUV-transparent, large bandgap crystals at CERN’s ISOLDE facility. Besides providing a highly precise value of the ^229m^Th excitation energy and a first estimate of the ionic lifetime, although to be corrected for in-medium effects, this experiment serves as proof-of-concept for the realization of a (multi-ion) solid-state nuclear clock, complementary to an ion-trap-based clock approach. With the ^229m^Th wavelength now constrained to $\lambda =$ 147.8(42) nm, developments for suitable lasers able to drive the clock transition have to focus on concepts like VUV frequency combs applying high harmonic generation (HHG) in noble-gas jets, due to the lack of intense, narrow-band (Hz–kHz) cw laser systems in this spectral regime. Bridging the gap until these lasers become available, broadband searches are conducted with existing laser systems, however with few GHz bandwidth, able to identify the nuclear resonance and constrain the search range for subsequent narrow-band frequency scans. An experimental platform has been set up at LMU in Munich, where a cyogenic Paul trap will be the centerpiece of a device able to generate, trap, and laser-manipulate ^229m^Th^q+^ ions for a measurement program that comprises the still awaited measurement of the (expected long) ionic lifetime of the thorium ions via hyperfine structure spectroscopy, realize their sympathetic laser cooling and finally couple, once becoming available in 2024, a VUV frequency comb laser to the stored and cooled ions for narrow-band resonance scans and ultimately for driving the nuclear clock transition in ^229^Th in a first prototype of a nuclear clock.

## Data Availability

Not applicable.
